# Removal of stabilizers from human serum albumin by adsorbents and dialysis used in blood purification

**DOI:** 10.1371/journal.pone.0191741

**Published:** 2018-01-24

**Authors:** Stephan Harm, Claudia Schildböck, Jens Hartmann

**Affiliations:** 1 Department for Health Sciences and Biomedicine, Danube University Krems, Krems, Austria; 2 Department of Pharmaceutical Technology and Biopharmaceutics, University of Vienna, Vienna, Austria; Kermanshah University of Medical Sciences, ISLAMIC REPUBLIC OF IRAN

## Abstract

**Introduction:**

Human serum albumin (HSA) is a monomeric multi-domain protein that possesses an extraordinary binding capacity. It plays an important role in storing and transporting endogenous substances, metabolites, and drugs throughout the human circulatory system. Clinically, HSA is used to treat a variety of diseases such as hypovolemia, shock, burns, hemorrhage, and trauma in critically ill patients. Pharmaceutical-grade HSA contains the stabilizers sodium caprylate and N-acetyltryptophanate to protect the protein from oxidative stress and to stabilize it for heat treatment which is applied for virus inactivation.

**Material and methods:**

The aim of this study was to determine if the two stabilizers can be depleted by adsorbent techniques. Several, adsorbents, some of them are in clinical use, were tested in batch and in a dynamic setup for their ability to remove the stabilizers. Furthermore, the removal of the stabilizers was tested using a pediatric high flux dialyzer.

**Results:**

The outcome of this study shows that activated charcoal based adsorbents are more effective in removal of N-acetylthryptophanate, whereas polystyrene based adsorbents are better for the removal of caprylate from HSA solutions. An adsorbent cartridge which contains a mix of activated charcoal and polystyrene based material could be used to remove both stabilizers effectively. After 4 hours treatment with a high flux dialyzer, N-acetyltryptophanate was totally removed whereas 20% of caprylate remained in the HSA solution.

## Introduction

Human serum albumin (HSA) is a protein consisting of 585 amino acids with a molecular mass of 66.7 kDa, is highly water soluble and has a strong negative charge. Albumin makes up half the normal intravascular protein mass, is synthesized in the liver and is responsible for 75–85% of the plasma colloid osmotic pressure [1]. The reference range for albumin concentrations in human blood is between 35 and 50 g/l in healthy individuals and the total intravascular mass is about 120 g. Albumin represents the most important plasma transport molecule in the human organism. It is responsible for the transport of hormones, fatty acids, amino acids (notably tryptophan and cysteine), steroids, metals (calcium, copper and zinc), numerous pharmaceutical drugs, bile acids, bilirubin and many others through the blood stream. The interaction and binding of these small molecules to HSA is important in determining their transport, distribution, metabolism and elimination in human body. Furthermore, albumin acts as important extracellular antioxidant and mediates protection from free radicals and other harmful chemical agents [2].

The structure and binding sites of the HSA molecule was first reported by He and Carter [3]. The heart-shaped protein consists of three homologous helical domains (I, II and III) whereas each domain is divided into two subdomains (IA, IB, IIA, IIB, IIIA, IIIB). While the domains have similar structure, each domain has different ligand-binding affinities and functions. Two most important and well characterized binding sites on human serum albumin are Sudlow sites I and II. Sudlow site I is located in subdomain IIA and Sudlow site II is located in subdomain IIIA [4]. It became evident from many ligand binding studies in the past that the principal binding regions on HSA are located in subdomains IIA and IIIA. Representative drugs which bind to site I are sulphonamides, coumarin anticoagulants and salicylate, to mention just a few [5]. The Sudlow site II is the most active binding site. Many ligands were found to bind preferentially to this site, for example ibuprofen and tryptophan [3, 6, 7]. As presented earlier [8], poor or strong binding to native HSA or infused pharmaceutical HSA is believed to affect half-life of drugs and, therefore, has an influence on their half-life and bioavailability in plasma. Due to the fact that pharmaceutical HSA shows significantly lower drug binding capacity compared to native HSA [9] and since many drugs are highly albumin-bound at therapeutic concentrations, drug pharmacokinetics may be influenced after large amounts of albumin infusion. Moreover, pharmaceutical HSA does not have the oxidative properties of native albumin [10]. In addition to its drug binding capabilities, HSA has also catalytic activities. One of the most investigated catalytic activities is the hydrolysis of acetyl salicylic acid to salicylic acid which is an esterase-like activity of the HSA molecule [11]. Because HSA can form a stable HSA-heme complex, the catalytic properties and ligand binding of HSA are strictly dependent on heme concentration in plasma which can be increased in haematological diseases [11]. Another major function of serum albumin is to protect from the toxic effects of bilirubin and other catabolic products of the human metabolism. Bilirubin is transported to the liver by albumin where it is transformed into a water soluble form by conjugation to glucuronic acid in order to enable the elimination via the bile into the duodenum. Other toxins which are shuttled by albumin through the blood stream to the liver for their detoxification are bile acids and certain amino acids. In disorders related to the liver, such as acute or chronic liver failure, cholestasis or genetic defects causing enzyme deficiencies, these liver toxins can accumulate in the patients’ blood up to 100 times higher than in healthy individuals. Based on this fact, the removal of lipophilic, albumin-bound substances such as bilirubin, bile acids, metabolites of aromatic amino acids, medium-chain fatty acids and cytokines should be beneficial to the clinical course of a patient in liver failure. This led to the development of filtration and absorption based blood purification devices. Although the use of extracorporeal liver support systems didn´t show an improvement of the 28 day survival rat they are used to prolong the survival time of the liver patient. However, it needs to be pointed out that without liver transplantation, the mortality is not decreased [12]. Another clinical application where albumin solutions were used is the therapeutic plasma exchange for sepsis treatment. Fresh frozen plasma, diluted with 5% albumin in different ratios are used to replace plasma loss during continuous flow plasmapheresis but none of the clinical studies which were carried out showed a beneficial outcome regarding survival rate [13–15].

Since in the case of liver diseases the production and detoxification of albumin by the liver is strongly reduced, albumin infusion is often part of the therapy. Also some extracorporeal blood purification systems for liver support therapy include albumin as detoxification agents in their circuits. These are the Molecular Adsorbent Recirculation System [16] (MARS) and the Single Pass Albumin Dialysis (SPAD) [17]. Further indications for which albumin therapy is considered include hypoalbuminemia, shock, hypovolemia, burns, surgery or trauma, acute respiratory distress syndrome, cardiopulmonary bypass and hemodialysis [18, 19] whereas albumin solution is mainly used in patients with cirrhosis who undergo paracentesis or are treated for hepatorenal syndrome. HSA for clinical application is separated from donated human plasma. Therefore, the risk of transmission of pathogenic viruses such as those causing hepatitis, HIV and others not yet identified exists. The viruses are usually heat-inactivated at 60°C for 10 hours. To avoid protein denaturation during this pasteurization process, caprylate and N-acetyl-tryptophanate (NAT) are widely used as stabilizers [20, 21]. Furthermore, caprylate ligand binding to the HSA molecule may protect HSA from aggregation during storage by increasing the electrical double layer that surrounds the protein [22]. However, the stabilizers are bound to Sudlow site II and therefore the transport function of pasteurized albumin is impaired. It is known from the literature that both stabilizers have additionally to their primary binding site in Sudlow site II other binding sites located on the protein with weak binding constants [23–26]. The common molar ratio for each of the two stabilizers is >5:1 (stabilizer: albumin). Because caprylate and NAT are metabolized in healthy humans, the use of high concentrations to stabilize albumin became state of the art until today but both stabilizers have been identified as vasodilators and may contribute to reduced renal perfusion [12]. Reduced albumin binding to drugs and other ligands can be observed in liver diseases with impaired liver functions. This is mostly the consequence of reduced albumin concentration and accumulation of drugs and metabolites (benzodiazepines, tryptophan, fatty acids, and bile acids) which are normally cleared by the liver [27–29]. It would be an advantage to remove the stabilizers from the HSA solution to administer albumin molecules with full native transport functions to patients. The Hepalbin adsorbent is a commercially available medical device which enables the bed-side removal of unwanted contaminations of human albumin solutions with caprylate and NAT and provides thereby a product which resolves the conflict between the need for stabilizers during production and storage and the desire for clean albumin with available binding sites after infusion or for albumin dialysis into liver disease patients.

The aim of this study was to determine if the industrial stabilizers can be depleted by adsorbent techniques. Several adsorbents were tested in batch and in a dynamic setup for their ability to remove the stabilizers and were compared to the commercially available Hepalbin filter. Additionally, the clearance of caprylate and NAT was evaluated with *in vitro* dialysis experiments.

## Material and methods

### Materials

For this study, two different human serum albumin solutions were used. The 200 g/L solution for infusion which contains 16 mmol/L sodium caprylate and 16 mmol/L N-acetyltryptophanate as stabilizers was obtained from Kedrion Biopharmaceuticals (Gallicano, Italy). The second one was a 97% lyophilisate without any stabilizers from Sigma Aldrich (St. Louis, Mo., USA). Bi-distillated water and physiological sodium chloride solution were purchased from Fresenius Kabi (Graz, Austria). Methanol, acetonitrile, water (all HPLC grade), n-acetyl-L-tryptophane, ethanol and phosphate buffered saline (PBS) were ordered from Sigma Aldrich (St. Louis, Mo., USA). Adsorbents which were used for this study and their providers are listed in Table 1.

**Table 1:** Adsorbents evaluated for their characteristics to remove caprylate and NAT from HSA.

adsorbent	provider	characteristics	clinical use	mean diameter [μm]
Prometh01	Fresenius Medical Care Adsorber Tec GmbH, Austria	polystyrene-divinylbenzene	plasma perfusion (Prometheus, liver support system)	550
diaMARS AC 250	Baxter, USA	activated charcoal	regenerate dialysate in MARS (liver support system)	900
Amberchrom CG161c	Dow Chemical, USA	polystyrene-divinylbenzene	no	120
Amberchrom CG161s	Dow Chemical, USA	polystyrene-divinylbenzene	no	35
Amberchrom HPR-10	Dow Chemical, USA	polystyrene-divinylbenzene	no	10
Hepalbin	Albutec GmbH, Germany	activated charcoal	remove stabilizer from pharmaceutical-grade HSA	filter plate
Hemosorba	Asahi Medical Co.Ltd, Japan	activated charcoal	hemoperfusion (liver support device)	650
Cytosorb	CytoSorbents GmbH, Germany	polystyrene-divinylbenzene coated with polyvinylpyrrolidone	hemoperfusion (sepsis and liver support device)	450

Albumin was measured with a Hitachi/cobas c311 automated analyzer with a corresponding test kit, both purchased from Roche (Penzberg, Germany). The adsorbents which were tested in this study are listed in Table 1.

### Adsorbent pre-treatment

Prior to use for adsorption experiments, the non-clinically used polystyrene-divinylbenzene (PS-DVB) based adsorbents (CG161c, CG161s and HPR-10) were washed with ethanol, water and 0.9% saline solution for 60 min each, using a ratio of one volume part adsorbent and four volume parts liquid for each washing step. This pre-treatment is necessary to wet the inner nano-porous surface of the dry adsorbent particles. Activated charcoals were washed with physiological sodium chloride solution.

### Removal of stabilizers in batch tests

The determination of the adsorption capacity for caprylate and N-acetyltryptophanate of each adsorbent was carried out in batch tests using 10% (w/v) of wet adsorbent (9 ml 20% HSA + 1 g adsorbent). Hepalbin, which is a charcoal based filter plate, was cut with a scalpel into small pieces with a maximum diameter of 2 mm. HSA without adsorbents was used as positive control and HSA without stabilizers was included as negative control in all batch tests. The incubation was carried out on a lab shaker for 24 hours at 37°C. Samples were taken after 0, 30, 60, 120, 240, 360 minutes and after 24 hours and were frozen at -20°C until quantification of caprylate, N-acetyltryptophanate, albumin and albumin binding capacity for Sudlow site II (ABiC II) was performed. To verify if the HSA without stabilizers contains some impurities which reduce the albumin binding capacity, additional batch tests were conducted where the HSA was incubated with Hemosorba and Prometh01.

### Removal of stabilizers in a dynamic model

**Fig 1:**
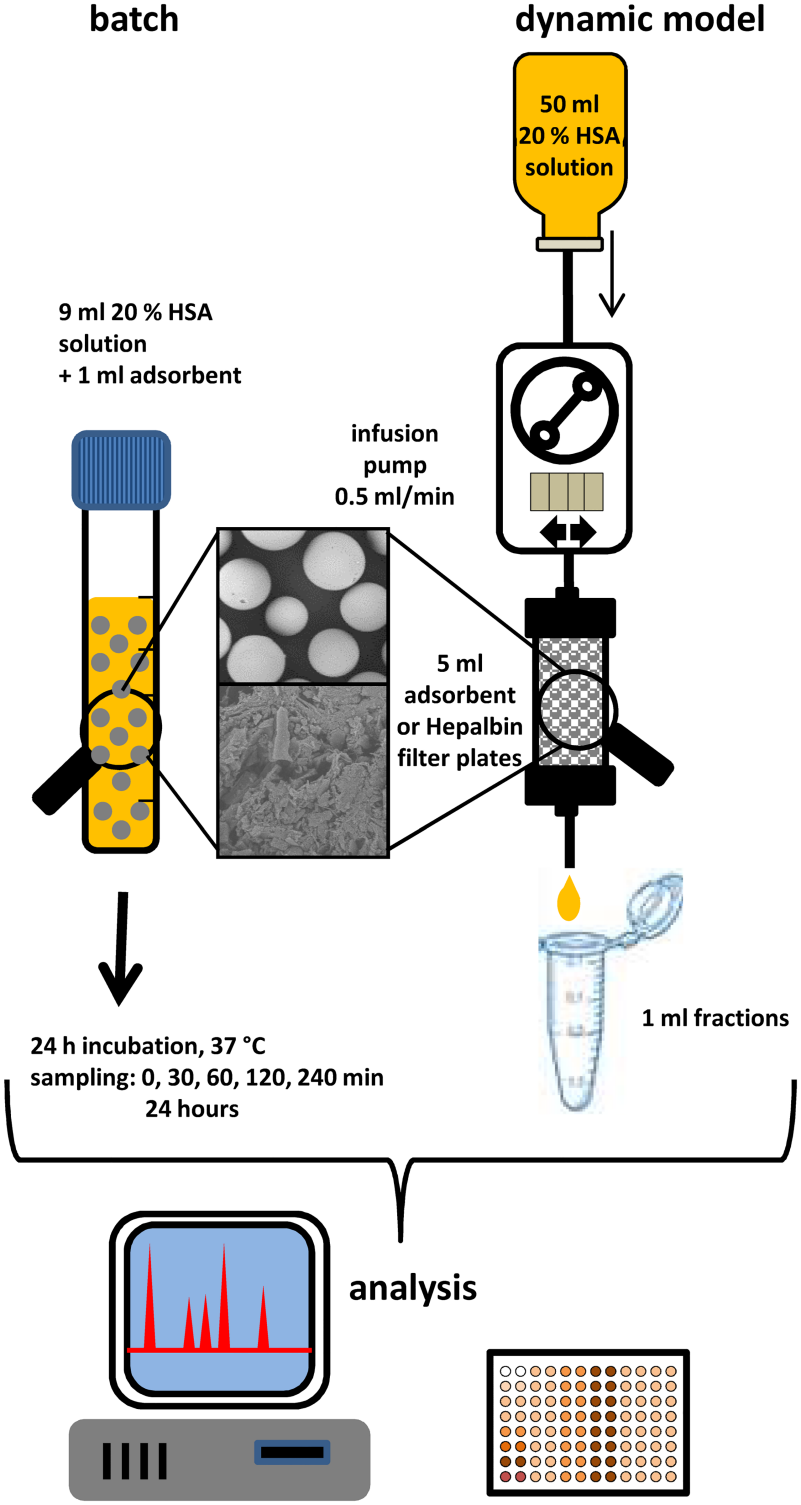
Schematic illustration of the batch tests and the dynamic model. The batch tests were performed with 10% (w/v) adsorbent in 20% HSA solution for 24 hours to determine the adsorption capacity of NAT and caprylate of each adsorbent. The dynamic model was used to simulate the removal rates of stabilizers from HSA solution before intravenous administration into the patient. 50 ml 20% HSA solution was pumped (0.5 ml/min) through a 5 ml adsorbent cartridge and 1 ml HSA fractions were collected post cartridge for determination of the NAT, caprylate level and ABiC.

To check if an adsorbent cartridge can be used during HSA infusion therapy for stabilizer depletion, a dynamic model was used (Fig 1). An empty cartridge (Rezorian, Sigma, St. Louis, Mo., USA) was packed with 5 ml of adsorbent material and flushed with 15 ml physiological sodium chloride solution. In case of the Hepalbin filter, circular plates with the same diameter as the cartridge were punched to fit them into the column. Afterwards, 50 ml 20% HSA solution was pumped through using a roller pump, with a flow rate of 0.5 ml/min. One milliliter fractions were collected post cartridge with a fraction collector Model 2110 (BIO-RAD, Hercules, California, USA). Every third fraction was used for quantification of caprylate, NAT, albumin and ABiC II.

### Removal of stabilizers by dialysis

**Fig 2:**
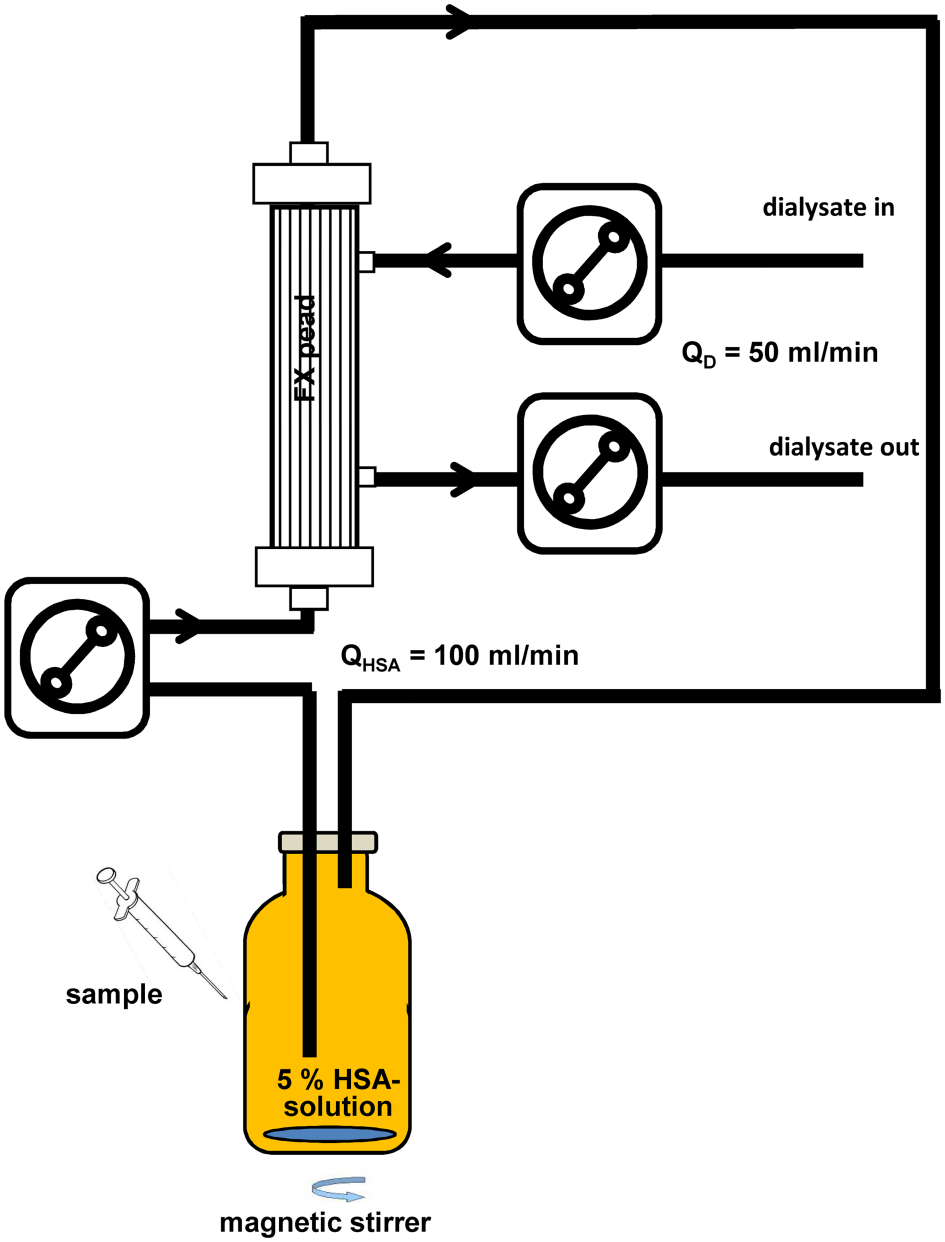
Setup of the dialysis circuit to determine the removal of the stabilizers by a high flux dialyser. 200 ml 5% HSA solution was dialysed against 0.9% NaCl solution using a pediatric high flux dialyser. Flow rates for the HSA solution was 100 ml/min and the dialysate flow was set to 50 ml/min. Samples were taken during 4 hours treatment time.

Dialysis experiments were carried out in miniaturized *in vitro* setups (Fig 2) using pediatric tubing and a polysulfone based pediatric high-flux dialyzers (FX paed, Fresenius Medical Care, Bad Homburg, Germany) with an effective membrane surface of 0.2 m^2^. 200 ml 5% HSA-solution was dialysed against 0.9% sodium chloride solution. The flow rates were *Q*_*HSA*_ = 100 ml/min for the HSA-solution and *Q*_*D*_ = 50 ml/min for the dialysis fluid. The experiments were carried out at 37°C and HSA-samples were taken after 0, 30, 60, 120, 180 and 240 minutes. Samples were stored at -20°C until quantification of caprylate, NAT, albumin and albumin binding capacity for Sudlow site II (ABiC II).

The clearance (C) was calculated using the formula

$$C=\frac{V}{t}\times \text{ln}\frac{c_{0}}{c_{t}}$$ (1)

where V = plasma volume [ml], t = treatment time [min], *C*_0_ = concentration before starting treatment [μM], *C*_*t*_ = concentration at time t [μM].

### Determination of the albumin binding capacity

The albumin binding capacity for binding site II (ABiC II) was estimated using the method described previously [29–31] with minor modifications. For the determination of the binding capacity on Sudlow´s site II, the fluorescent dye Dansylsarcosine (DS) (Sigma Aldrich, St. Louis, Mo., USA) was used. The binding of DS to the albumin molecule on Sudlow´s site II leads to an increase of the fluorescence intensity of the chromophore. Consequently, albumin without stabilizers results in a stronger fluorescence signal since it is able to bind higher amounts of DS. For ABiC II measurement a 10 mM DS stock solution, solved in acetonitrile, was prepared and stored in the dark at 4°C. Immediately before usage a 1 mM DS working solution, diluted with 10 mM PBS was prepared. The measurements were performed in 96-well plates (cellstar, PS, F-bottom, μclear, black) purchased from Greiner Bio-One GmbH (Kremsmünster, Austria). 100 μl of each 0.1 mM albumin sample was mixed with 10 μl DS working solution and the fluorescence signal was measured with a multi detection microplate reader (BioTec Instruments Inc., Winooski, VT, USA; excitation wavelength 360 nm, emission wavelength 460 nm). The binding capacity was calculated in percent, where the stabilizer-free HSA from Sigma was set to 100% (positive control), according to the following equation:

$$ABiCII(\%)=\frac{\begin{array}{ccccc} fluorescence & of & adsorbent & treated & HSA \end{array}}{\begin{array}{ccccc} fluorescence & of & HSA & without & stabilizer \end{array}}\times 100$$ (2)

As negative control, the untreated 20% HSA-solution for infusion was used. Both controls were included in each analytical run. The albumin concentration of each sample was checked with the Hitachi/cobas 311c automated analyser. Each sample was diluted with physiological sodium chloride solution to yield a 0.1 mM albumin solution and a molecular weight of 66400 Da for albumin was assumed.

### Determination of N-acetyltryptophanate level

The quantification of N-acetyltryptophanate was performed with a high performance liquid chromatography method. Samples were precipitated by adding 50 μl HSA sample to 250 μl methanol, vortexed, and cooled at -80°C for 20 minutes. After centrifugation (14000 g, 5 min) 20 μl of the supernatant was injected into the HPLC column (150 x 4.6 mm Nucleosil 100–5 C18 column combined with a 4 x 3 mm Nucleosil 100–5 C18 guard column; Macherey-Nagel, Düren, Germany). The temperature of the column oven was set to 35°C. The elution consisted of a linear gradient program from 30 to 50% methanol in water over 6.5 minutes, maintained at 50% methanol for 1 minute and returned to 30% methanol for 1 minute. The flow rate was 0.5 ml/min and absorbance detection at 280 nm was performed for NAT quantification. The amount of N-acetyltryptophanate was calculated in percent of the untreated 20% HSA-solution (Kedrion Biopharmaceuticals, Italy), and as negative control the stabilizer-free HSA (Sigma Aldrich, USA) was analyzed. The recovery of NTA using this HPLC procedure is 72.3 ± 0.5% (S1 Fig). It was determined by comparing the peak areas between NTA diluted in methanol and NTA spiked in stabilizer-free albumin solution.

### Determination of caprylate level

The quantification of caprylate was performed with a free fatty acid quantification assay kit (abcam, Cambridge, UK). This kit provides a sensitive, enzyme-based method for colorimetric detection of long- and middle chain free fatty acids in biological samples. Concentrations of free fatty acids in the samples were calculated from standards which are included in the kit. To obtain a signal in the standard range (0 to 200 μM), the samples were diluted 1:100 with dilution reagent which was provided with the kit. The amount of caprylate was calculated in percent of the untreated 20% HSA-solution (Kedrion Biopharmaceuticals, Italy), and as negative control the stabilizer-free HSA (Sigma Aldrich, USA) was used.

### Statistics

All experiments were carried out at least thrice and data are expressed as mean ± SD which was calculated using Microsoft Excel 2010. Significances were calculated with the one-tailed t-test using SigmaPlot for Windows Version 13.0. Area under the curve and area above the curve calculations were conducted with Microsoft Excel for Windows 2010.

## Results and discussion

### Removal of stabilizers in batch tests

**Fig 3:**
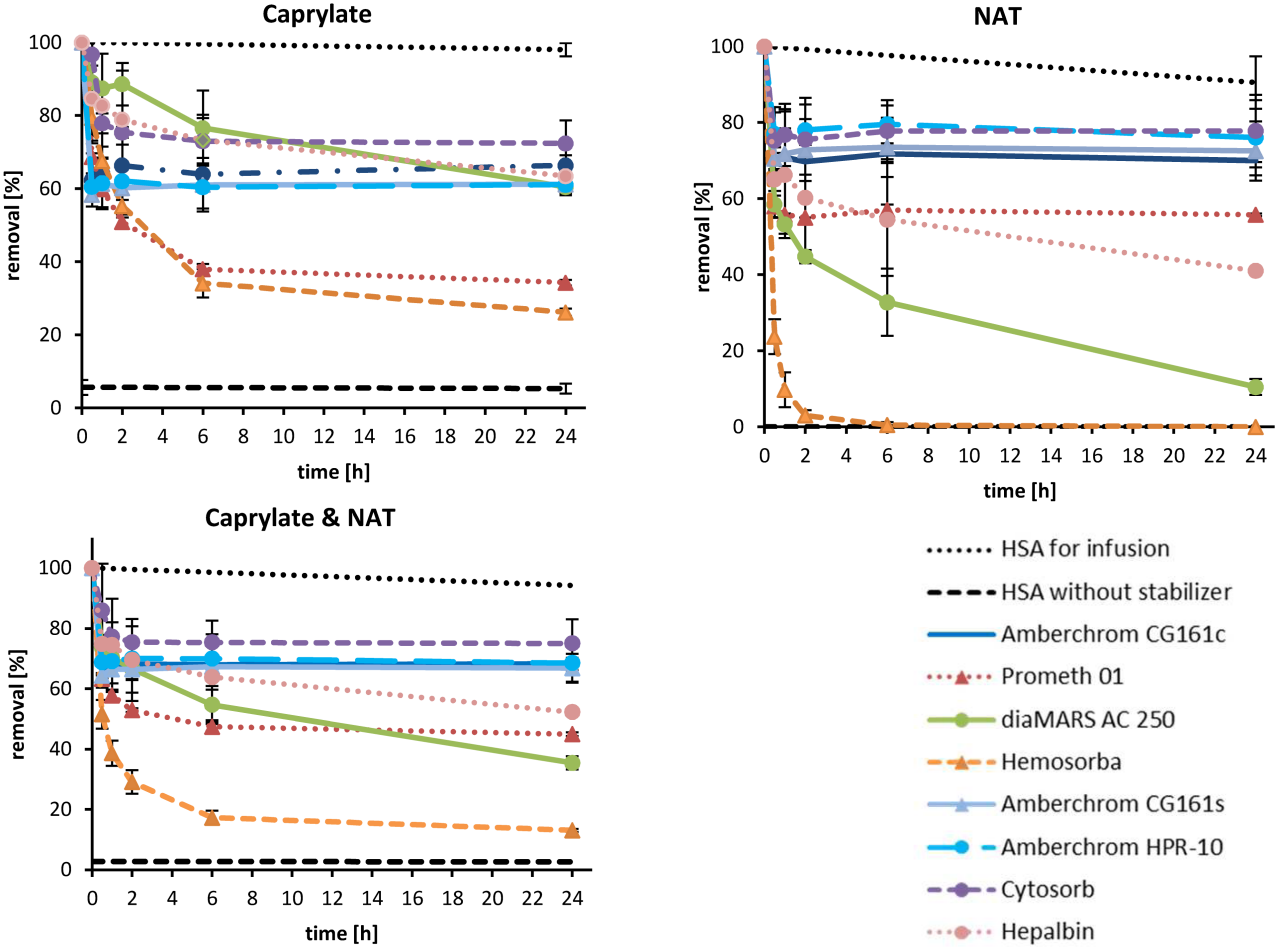
Percental removal rates of caprylate, N-acetyltryptophanate and of both from 20% HSA infusion solution by different adsorbents tested in batch. HSA solution was incubated with 10% (v/v) adsorbent for 24 hrs at 37°C. Samples for NAT and caprylate quantification were taken after 30, 60, 120, 240, 360 min and 24 hrs. The stabilizer levels of HSA solution without adsorbent (dotted line) were set to 100%. The caprylate and NAT levels of stabilizer free lyophilized HSA act as negative control (dashed lines).

The adsorption capacity of different adsorbents regarding caprylate and NAT removal was tested in batch. The results show that larger adsorbent particles (diaMARS AC 250, Cytosorb, Prometh01 and Hemosorba) reach the equilibrium concentration after several hours compared to the smaller adsorbent particles (CG161c, CG161s, HPR10) which show very fast adsorption kinetics (Fig 3). The maximum time (t_max_) which was needed to reach an equilibrium concentration for caprylate and NAT and the adsorption capacity (mg caprylate or NAT per g adsorbent) for the two stabilizers in the batch tests are listed in Table 2. Particle size and pore size are geometric parameters of the adsorbent material which influence the adsorption kinetic of target molecules. The outer pores of the adsorbent surface act as a molecular sieve and prevent the entry of molecules that are larger than the molecular cut-off of the pores. In case of adsorbent materials with defined pore structure, the viscosity radius of molecules, also known as hydrodynamic radius, is essential for adsorption because it determines if the molecule can come in contact with the inner adsorbents surface or not [32]. A high dependence of the diffusion time (*t*_*Δx*_) on the diffusion path (Δx) can be found using the Einstein-Smoluchowski equation, which predicts that:

$$t_{\Delta x}=\frac{\Delta x^{2}}{2D}$$ (3)

where D is the diffusion coefficient. Consequently, smaller adsorbent particles show faster adsorption kinetics.

**Table 2:** The maximum time [h] to reach the equilibrium concentration for caprylate and NAT removal and adsorption capacity [mg/g] for caprylate and NAT of each adsorbent in the batch tests.

	t_max_ to reach the equlibrium for caprylate [h]	t_max_ to reach the equlibrium for NAT [h]	adsorption capcity [mg caprylate /g adsorbent]	adsorption capacity [mg NAT /g adsorbent]
Amberchrom CG161c (120 μm)	0.5	0.5	7.3	10.6
Prometh01	> 24	1	14.2	15.7
diaMars AC 250	> 24	> 24	8.5	31.7
Hemosorba	> 24	24	15.9	35.5
Amberchrom CG161s (35 μm)	0.5	0.5	8.4	9.8
Amberchrom HPR-10	0.5	0.5	8.4	8.5
Cytosorb	6	0.5	6.0	7.9
Hepalbin	> 24	> 24	7.9	20.9

Fig 3 shows that NAT is removed more efficiently by charcoal based adsorbents (diaMARS AC 250, Hepalbin and Hemosorba) compared to polystyrene based adsorbents. Only Hemosorba was able to remove all NAT from the HSA solution. A possible reason for this could be that Hemosorba has, compared to the other charcoal based adsorbents (diaMARS AC 250. Hepalbin) the largest accessible inner pore surface. From the polystyrene based adsorbent only Prometh01 shows good NAT adsorption properties (44 ± 1%). The adsorbents which show the highest caprylate removal are Hemosorba (73 ± 3%) and Prometh01 (66 ± 1%). Because both stabilizers were added in the same molar ratio (16 mM), we were able to calculate the mean removal rate for both stabilizers (Fig 3). The ranking regarding removal rate of both stabilizers reads as follows: Hemosorba (87 ± 1%) > diaMARS AC 250 (65 ± 2%) > Prometh01 (55 ± 1%) > Hepalbin (48 ± 1%) > CG161c (32 ± 2%) > CG161s (33 ± 5%) > HPR-10 (32 ± 6%) > Cytosorb (25 ± 8%).

**Fig 4:**
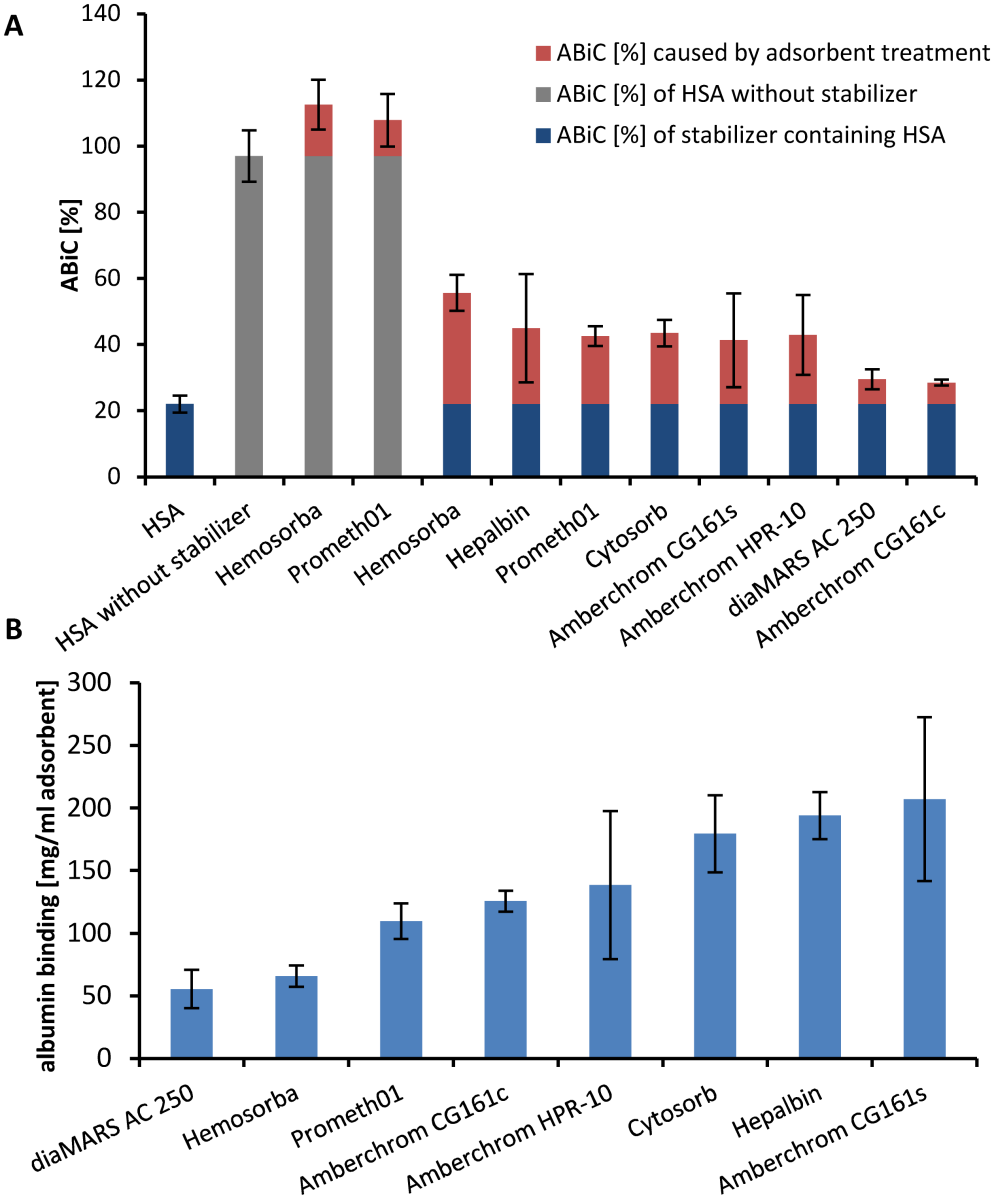
The adsorbent treatment increases the ABiC II levels. The albumin binding capacity (ABiC II) after adsorbent treatment was compared to untreated HSA and HSA without stabilizers, which was set to 100% (A). All adsorbent treatments caused a significant (p ≤ 0.05) increase of ABiC II. The albumin concentration was measured after 24 hours adsorbent treatment in batch to calculate the albumin binding in mg per ml adsorbent volume (B).

Albumin binding capacity for binding site II (ABiC II) is a simple method for characterizing the site II-specific binding functions of the albumin molecule [33]. After diluting the different albumin samples from the batch tests to the same albumin concentration (0.1 mM) and incubation with the binding site II-specific fluorescent marker, the amount of bound marker can be determined by fluorescence detection. The ABiC II of HSA without any stabilizer was set to 100% binding capacity. As it is shown in Fig 4A the binding capacity of commercially available HSA for infusion is highly reduced by the stabilizers (26 ± 7%) compared to the HSA without stabilizer. The results indicate that the increase of ABiC II correlates more with the removal of caprylate than with NAT. Hemosorba was able to raise the ABiC II value from 26 ± 7% to 57 ± 7% whereas the CG161c treatment caused only an increase of 2 ± 1%. All adsorbent treatments caused a significant (p ≤ 0.05) increase of ABiC II. Also the ABiC II of the stabilizer free albumin was increased using Hemosorba (116 ± 1%) and Prometh01 (111 ± 1%). This indicates that the stabilizer free HSA includes some impurities which influence the ABiC II and can be removed by adsorption treatment (Fig 4). The albumin level was determined to evaluate the influence of each adsorbent on the albumin level of the HSA solution after treatment. The highest loss of albumin was caused by CG161s treatment which shows an albumin binding of 210 mg per ml adsorbent. With the exception of Hepalbin, the charcoals showed low albumin adsorption whereas the lowest reduction on the albumin level was achieved by the diaMARS AC 250 adsorbent (Fig 4B).

### Removal of stabilizers in the dynamic model

**Fig 5:**
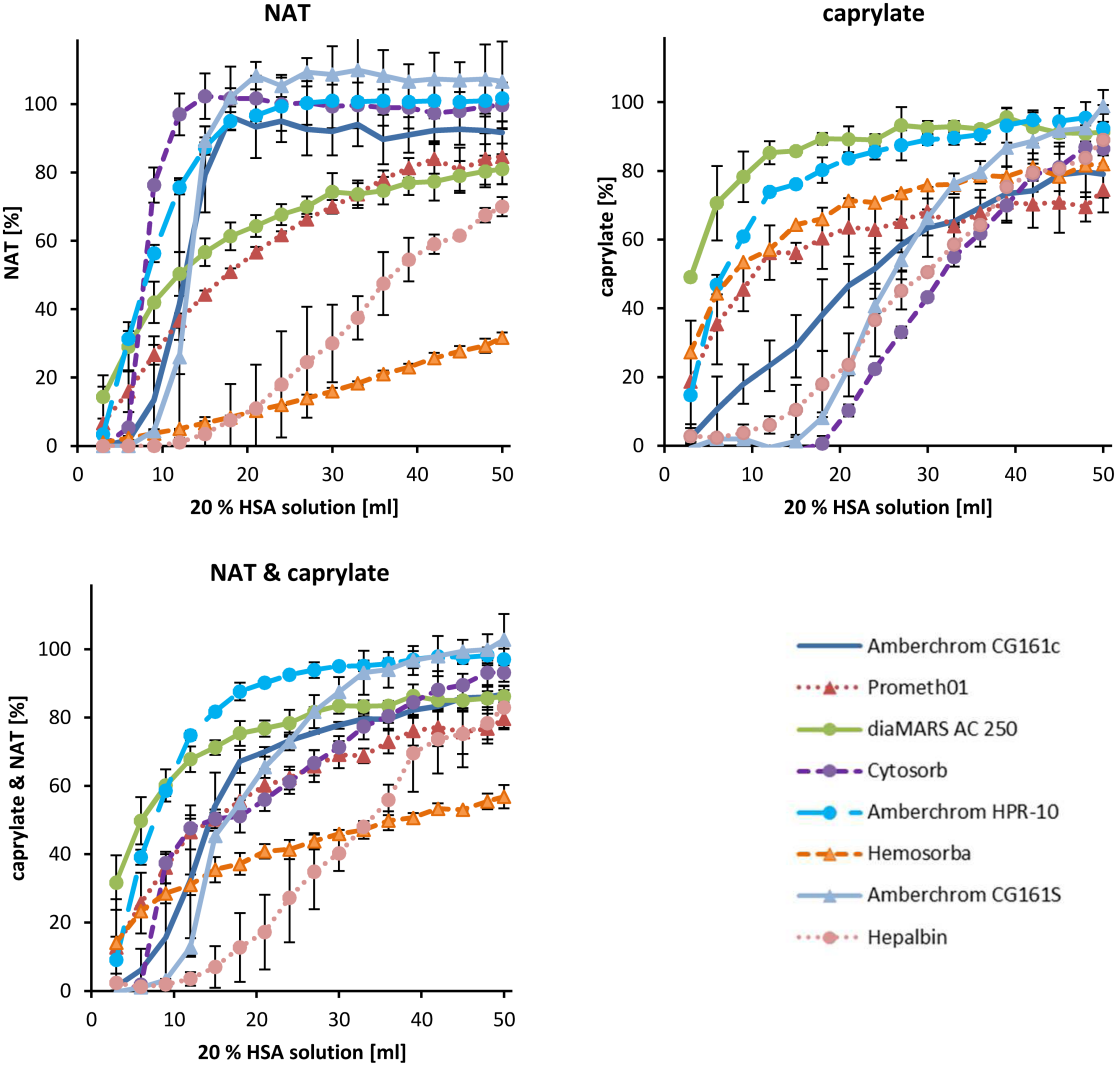
Pre cartridge concentrations of stabilizers from 20% HSA solution using different adsorbents in the dynamic model. 50 ml 20% HSA solution was pumped with 0.5 ml/min through a 5 ml adsorbent filled cartridge. 1 ml fractions were collected post cartridge for NAT and caprylate measurement.

For the practical application, the adsorbents were tested in a dynamic model, where the HSA solution for infusion was pumped through a cartridge filled with adsorbent and the removal rate of stabilizers was measured downstream from the cartridge. The aim of this setup was to simulate an intravenous HSA infusion where the HSA is depleted of stabilizers using an adsorption column before it is infused into the blood stream (Fig 1). To save material and costs, the model was downscaled using 50 ml 20% HSA solution which was pumped in single pass through a 5 ml adsorbent filled cartridge. Based on the flow rate of 0.5 ml/min, the contact time of HSA to the adsorbent material was calculated to be 10 min. The relatively short contact time between adsorbent surface and the HSA solution, results in a shorter diffusion distance. Consequently, large adsorbent particles are not as efficient in the dynamic model compared to the batch test where the contact time was 24 hours. To illustrate that the particle size of adsorbents influences the outer adsorbent surface we calculated the outer adsorbent surface of a 5 ml filled cartridge. Assuming that all adsorbent are spherical particles and follows the close-packing of equal spheres (adsorbent volume = 74% of cartridge volume) the outer adsorbent surface of HPR-10 was about 100 times larger than that of the diaMARS AC in the dynamic model (Table 3). As it is shown in Fig 3, the post cartridge level of stabilizers is very low in the first fractions and increases depending on the adsorbent. For mathematical evaluation of the dynamic model, the adsorbed amount of NAT and caprylate is shown as area above the curve in Fig 5. The level of removed stabilizers is shown in Table 4. In summary, it can be stated that in the dynamic model the Hepalbin (-60%) and Hemosorba (-60%) are the most effective adsorbents for both stabilizers. From the polystyrene based adsorbents, Prometh01 (-40%) and Cytosorb (-38%) showed the best removal rates of stabilizers in the dynamic model, whereby Cytosorb showed the highest efficiency regarding caprylate removal (Fig 5).

**Table 3:** Calculated outer adsorbent surface of an 5 ml filled cartridge, assuming that all adsorbent are spherical particles and follows the close-packing of equal spheres (adsorbent volume = 74% of cartridge volume).

	particle diameter [μm]	particle volume [cm^3^]	particle/ cartridge	outer surface/ particle [cm^2^]	outer particle surface/ cartridge [cm^2^]
diaMARS AC 250	900	3.82 x 10^−4^	0.97 X 10^4^	2.5 x 10^−2^	247
Hemosorba	650	1.44 x 10^−4^	2.57 x 10^4^	1.3 x 10^−2^	342
Pormeth01	550	8.71 x 10^−5^	4.25 x 10^4^	9.5 x 10^−3^	404
Cytosorb	450	4.77 x 10^−5^	7.75 x 10^4^	6.4 x 10^−3^	667
Amberchrom CG161c	120	9.05 x 10^−7^	4.09 x 10^6^	4.5 x 10^−4^	1850
Amberchrom CG161s	35	2.24 x 10^−8^	1.65 x 10^8^	3.8 x 10^−5^	6343
Amberchrom HPR-10	10	5.24 x 10^−10^	7.07 x 10^9^	3.1 x 10^−6^	22200

**Table 4:** ABiC II values compared to HSA without stabilizer in percent and removal rates of caprylate, N-acetyltryptophan and both in percent from a 20% HSA solution using different adsorbent in the dynamic model.

	Hepalbin	Cytosorb	CG161s (35 μm)	Hemosorba	CG161c (120 μm)	Prometh 01	diaMARS AC 250	HPR-10
*removal NAT [%]*	67 ± 11	11 ± 6	15 ± 4	85 ± 1	24 ± 3	40 ± 2	29 ± 16	19 ± 2
*removal caprylate [%]*	51 ± 12	64 ± 1	53 ± 2	31 ± 5	51 ± 5	40 ± 7	13 ± 2	13 ± 1
*removal NAT & caprylate [%]*	59 ± 12	38 ± 3	34 ± 3	59 ± 3	38 ± 4	40 ± 5	21 ± 9	16 ± 1
*albumin loss [%]*	3 ± 1	4 ± 1	4 ± 1	1 ± 1	9 ± 3	3 ± 1	3 ± 3	5 ± 1
*ABiC II [%]after treatment*	82 ± 24	59 ± 1	73 ± 5	44 ± 5	39 ± 3	25 ± 1	44 ± 16	26 ± 1

**Fig 6:**
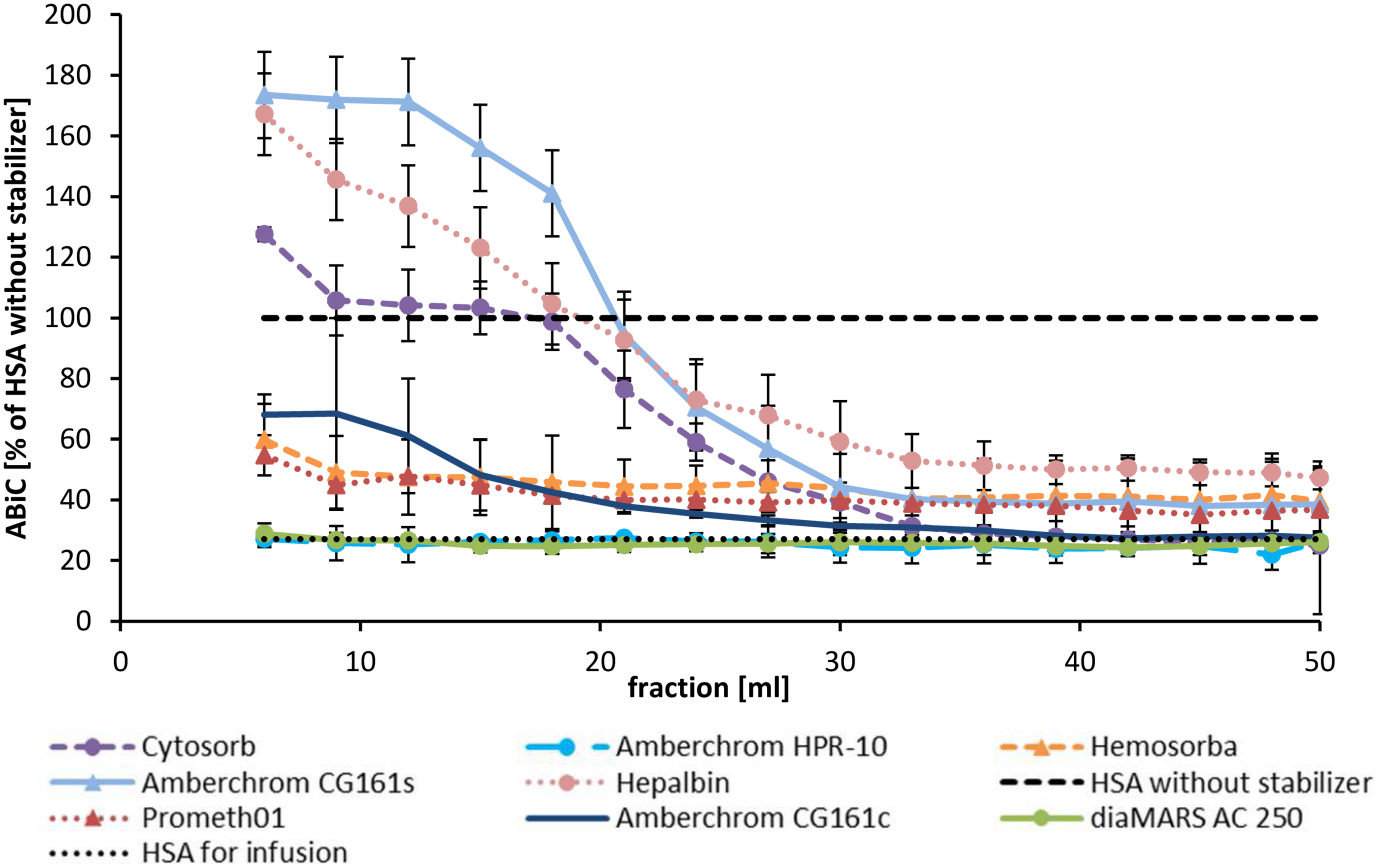
ABiC II levels of the post cartridge fractions from the dynamic model. ABiC II was determined from post adsorbent cartridges fractions. ABiC II of HSA without stabilizer (dotted line) was set to 100%. ABiC II of untreated HSA is shown in broken line.

Due to the fact that the increase of ABiC II correlates more with the removal of caprylate than with the removal of NAT, the highest ABiC II values in the dynamic set up could be reached using Hepalbin and CG161s whereas only little increase of ABiC II was observed using diaMARS AC 250 or HPR10 adsorbent (Table 1 and Fig 6). The reason for this finding is that NAT has a lower binding affinity to albumin than DS and, therefore, DS will bind to albumin independently from the NAT concentration. As a consequence, the ABiC II method is only suitable for strongly albumin-bound substances.

The quantification of ABiC II in the dynamic model was determined by calculating the area under the curve of Fig 6. The data are given in percent compared to HSA without stabilizer (Table 4). The results of the ABiC II determination show that the first post adsorbent fractions of Hepalbin, Cytosorb and CG161s achieve a higher ABiC II level than compared to HSA without stabilizers. This might indicate that also the HSA without stabilizer has not the full binding capacity and that the binding site II is occupied with some impurities originating from the manufacturing process. This was proven by the performed batch tests where we could show that the ABiC II of stabilizer free HSA increased after adsorbent treatment (Fig 4 and S2 Fig). The highest calculated ABiC II using the dynamic model was achieved using the Hepalbin adsorbent (82%) followed by CG161s (74%) and Cytosorb (59%). HPR10 and diaMARS AC 250 treatment caused the lowest increase of ABiC II. By comparing the removal rates of NAT and caprylate to the determined ABiC II it can be concluded that caprylate removal has more influence on the improvement of the ABiC II than the removal of weakly bound NAT.

### Removal of stabilizers by dialysis

**Fig 7:**
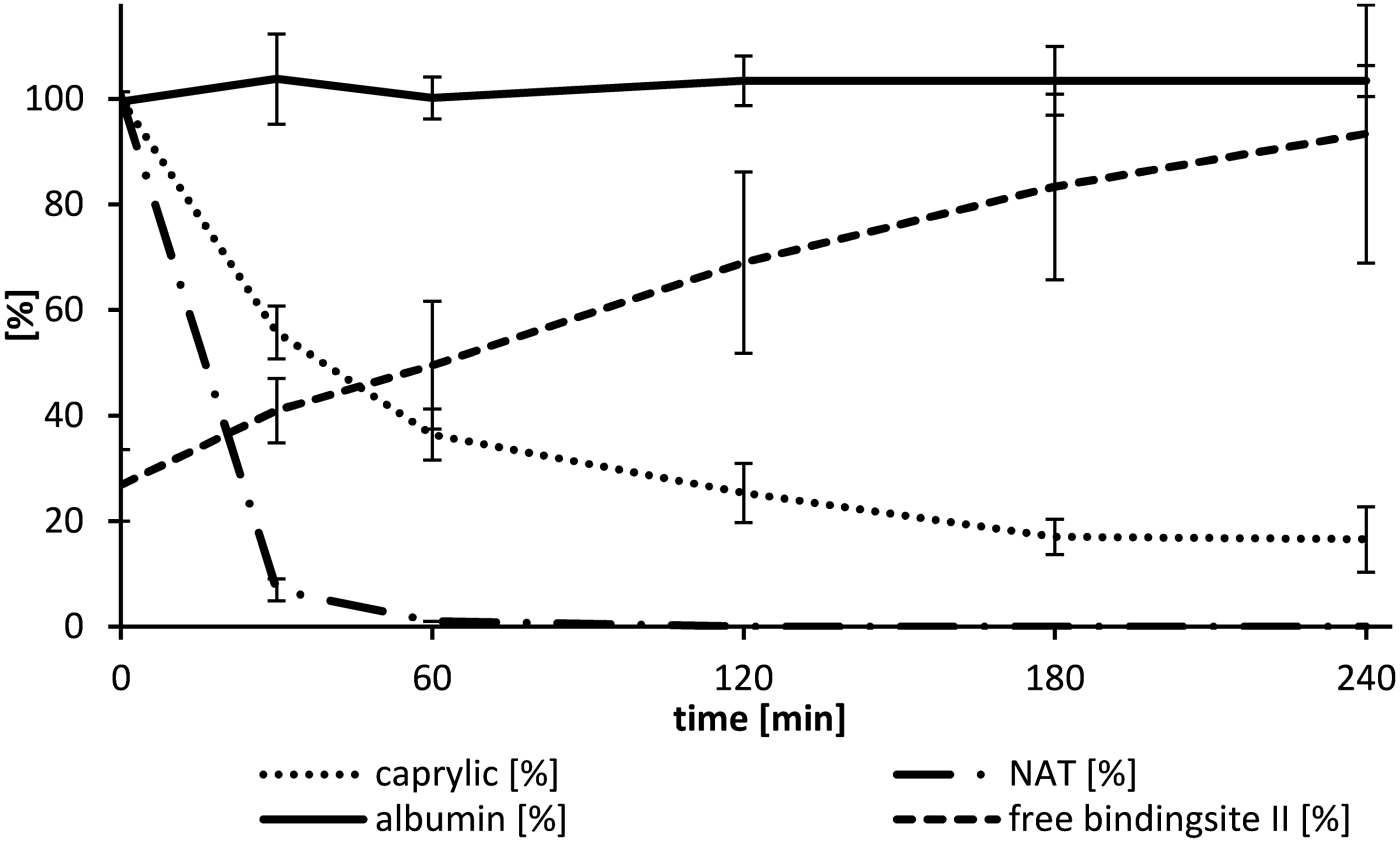
Removal of stabilizers during 4 hours dialysis. 200 ml 5% HSA solution was dialyzed against physiological sodium chloride solution using a pediatric high-flux dialyzer. The flow rate of the HSA solution was 100 ml/min and the dialysate flow was set to 50 ml/min. The treatment time was 4 hours and samples were taken after 30, 60, 120, 180 and 240 min, n = 4.

Albumin infusion is often applied in patients who suffer from liver failure. In some extracorporeal liver support systems such as MARS and Prometheus, a dialyzer is included to remove uremic toxins from patient’s blood. Water soluble toxins as well as weakly albumin bound toxins can be removed by dialysis. To evaluate if the two stabilizers can be removed by dialysis, a downscaled dialysis setup was used, where 200 ml 5% HSA solution was dialyzed against physiological sodium chloride solution for 4 hours (Fig 2). Since the binding constant between HSA and caprylate (K_a_ ≤ 5.5 x 10^5^) [20, 34] is higher than between HSA and NAT (tryptohan: Ka ≤ 1 x 10^4^), NAT is removed easier by dialysis because of the higher rate of unbound molecules (Fig 2). In our dialysis setup, NAT was totally removed after 60 min, whereas caprylate could be lowered after 4 hours to 17 ± 6% compared to the starting concentration (Fig 7). A 93 ± 24% recovery of the ABiC II was achieved by dialysis and a reduction of the albumin level was not observed which can be explained by the low sieving coefficient for albumin which is given < 0.001 for the FX paed in the product data sheet. The calculated clearance according to equation [1] is listed in Table 5. It is noticeable that the caprylate clearance decreases from 4 ± 1 at time point 30 minutes to 0.2 ± 0.6% after 4 hours. These results show that the binding sites for the five caprylate molecules have varying binding constants. At a molar ratio of one (HSA: caprylate) the removal rate of caprylate via dialysis reaches nearly zero which means that one caprylate binding site on the HSA molecule is comparably strong as the bilirubin binding site (K_a_ = 3 x 10^6^ M^-1^).

**Table 5:** Calculated clearance rates using the formula (1) for NAT and caprylate from the dialysis experiment (n = 4).

*time [min]*	*C*_*NAT*_ *[%]*	*C*_*caprylate*_ *[%]*
30	18 ± 2	4 ± 1
60	15 ± 0	3 ± 1
120	N/A	1.2 ± 0.3
180	N/A	1.5 ± 0.3
240	N/A	0.2 ± 0.6

## Conclusions

For patients suffering from hypoalbuminemia, hyperbilirubinemia or other illnesses causing an impaired transport capacity, the removal of the albumin-blocking compounds is of high importance. The same is true for patients who need albumin substitution. The removal of stabilizers from HSA could be achieved by using an adsorbent cartridge where the HSA solution passes through before it is administered intravenously to the patient, similar to the Hepalbin filter. The study shows that activated charcoals have better adsorption properties for NAT, whereas polystyrene based adsorbents are more suitable for caprylate removal. Because most of the pharmaceutical grade HSA includes NAT and caprylate in equal molar ratios, a combination of these two materials would be favourable to efficiently remove both stabilizers. The results from the dynamic setup revealed that the use of Hepalbin filter caused the highest ABiC in Sudlow site II and is an efficient medical device for applying stabilizer reduced albumin infusion into the patient. We could further show that all NAT and more than 80% of caprylate can be removed by dialysis. These results suggest that an albumin therapy for liver patients could be combined with dialysis treatment to achieve stabilizer reduced albumin with almost completely restored transport capacity and, therefore, detoxifying capability. A combination of dialysis with an adsorbent based blood purification system, such as Prometheus or MARS, is able to reduce the level of stabilizers of human serum albumin. This implies that the albumin infusion should be given shortly before or at the beginning of the extracorporeal liver support treatment to reduce the level of stabilizers in patients’ blood. Furthermore, the results confirm previous studies which showed that the Open Albumin Dialysis system (OPAL), which uses several Hepalbin filters in the dialyse circuit instead of the diaMARS AC 250 charcoal in the MARS system, is an efficient system to generate high albumin binding capacity in liver failure patients [35, 36].

## Supporting information

S1 Fig Recovery of the HPLC method for NAT quantification. The recovery was determined by comparing the peak areas between NTA diluted in methanol and NTA spiked in stabilizer-free albumin solution. The tested NAT concentrations were 4 and 16 mM. All samples for HPLC injection were done in triplicates.
(DOCX)

S2 Fig Adsorbent treatment of stabilizer free HSA increase the ABiC II value. To verify if the HSA without stabilizers contains some impurities which reduce the albumin binding capacity, additional batch tests were conducted where the HSA was incubated with Hemosorba and Promth01.
(DOCX)
