# Supplementary material for: Removal of stabilizers from human serum albumin by adsorbents and dialysis used in blood purification
Source: PLoS One. 2018 Jan 24;13(1):e0191741. doi: 10.1371/journal.pone.0191741 (PMC5783404; doi:10.1371/journal.pone.0191741)
Supplement: S1 Fig — The recovery was determined by comparing the peak areas between NTA diluted in methanol and NTA spiked in stabilizer-free albumin solution. The tested NAT concentrations were 4 and 16 mM. All samples for HPLC injection were done in triplicates. (DOCX) [file pone.0191741.s001.docx]

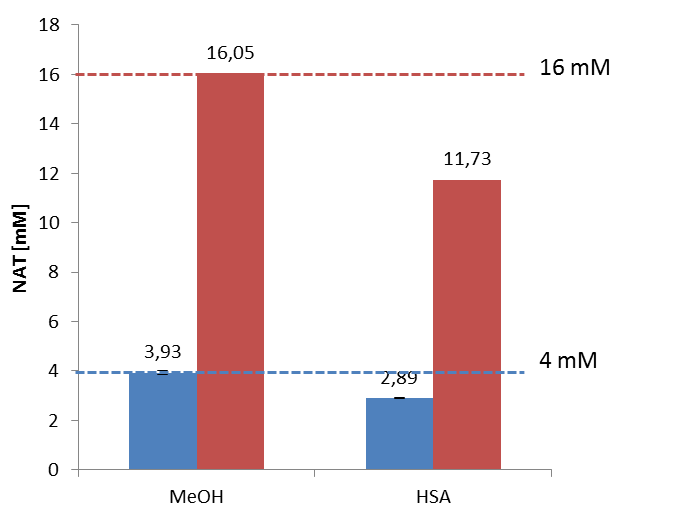


S1 Fig. Recovery of the HPLC method for NAT quantification. The recovery was determined by comparing the peak areas between NTA diluted in methanol and NTA spiked in stabilizer-free albumin solution. The tested NAT concentrations were 4 and 16 mM. All samples for HPLC injection were done in triplicates. The determined NAT recovery of the HPLC method is 72.3 ± 0.5 %.
