# Supplementary material for: Removal of stabilizers from human serum albumin by adsorbents and dialysis used in blood purification
Source: PLoS One. 2018 Jan 24;13(1):e0191741. doi: 10.1371/journal.pone.0191741 (PMC5783404; doi:10.1371/journal.pone.0191741)
Supplement: S2 Fig — To verify if the HSA without stabilizers contains some impurities which reduce the albumin binding capacity, additional batch tests were conducted where the HSA was incubated with Hemosorba and Promth01. (DOCX) [file pone.0191741.s002.docx]

S2 Fig. Adsorbent treatment of stabilizer free HSA increase the ABiC II value. To verify if the HSA without stabilizers contains some impurities which reduce the albumin binding capacity, additional batch tests were conducted where the HSA was incubated with Hemosorba and Promth01.
